# Pine Forest Plantations in the Neotropics: Challenges and Potential Use of Ectomycorrhizal Fungi and Bacteria as Inoculants

**DOI:** 10.3390/jof11050393

**Published:** 2025-05-20

**Authors:** Yajaira Baeza-Guzmán, Sara Lucía Camargo-Ricalde, Dora Trejo-Aguilar, Noé Manuel Montaño

**Affiliations:** 1 Doctorado en Ciencias Biológicas y de la Salud, Universidad Autónoma Metropolitana, Mexico City, Mexico; ybaezaguzman@gmail.com; 2 Departamento de Biología, División de Ciencias Biológicas y de la Salud, Universidad Autónoma Metropolitana, Unidad Iztapalapa, Mexico City 09310, Mexico; slcr@xanum.uam.mx; 3 Facultad de Ciencias Agrícolas, Universidad Veracruzana, Circuito Gonzalo Aguirre Beltráns/n, Zona Universitaria, Xalapa 91090, Mexico

**Keywords:** diversity, *Pinus*, reforestation, seedling quality, sustainable forest management

## Abstract

Forest plantations in the Neotropics aim to alleviate pressure on primary forests. This study synthesizes knowledge on pine species used in these plantations, emphasizing the challenges and potential of ectomycorrhizal fungi and bacteria as inoculants. An analysis of 98 articles identifies 23 pine species in Mexico and Central America and about 16 fast-growing species in South America. While pine plantations provide a habitat for generalist species, they reduce the richness of specialist species. Ectomycorrhizal fungi and bacterial diversity in plantations with introduced pines is up to 20% lower compared to native ecosystems. *Suillus* and *Hebeloma* are commonly used as mycorrhizal inoculants for Neotropical and introduced species, including *Pinus ponderosa* and *Pinus radiata* in South America. Commercial inoculants predominantly feature the fungal species *Pisolithus tinctorius*, alongside bacterial genera such as *Bacillus*, *Cohnella*, and *Pseudomonas*. This study emphasizes the importance of leveraging native microbial communities and their synergistic interactions with ECM fungi and bacteria to enhance seedling growth and quality. Such a combined approach can improve plantation survival, boost resilience to environmental stressors, and promote long-term productivity. These findings underscore the need to incorporate native fungi and bacteria into inoculant strategies, advancing sustainable forestry practices and ecosystem adaptation in the Neotropics.

## 1. Introduction

The genus *Pinus* L. (Pinaceae) is globally significant, with Mexico, Central America, and the Caribbean hosting the highest diversity of pine forests, including 45% of the total species [1]. The Neotropics, particularly Mexico and Central America, are crucial for *Pinus* biodiversity, with 23 endemic species and a significant concentration of mid- to low-latitude diversity [2]. Moreover, approximately 20% of the pine species have been introduced beyond their native ranges of distribution [3].

Recently, based on the expansion patterns of pines, Morrone et al. proposed a biogeographical regionalization area in the Neotropics that spans from and trough the Antilles and Bahamas, Central and Southern Mexico, Central America, Northwest–Southeast of South America, the Andean highlands, desert areas along the coast of Peru and Northern Chile to Central-Western Argentina [4]. Within this huge area, 68 pine species have been reported, representing ca. 62% of the total pine species worldwide [1]. However, these species can be native or introduced and have primarily been utilized for commercial purposes.

Currently, forest plantations are presented as an alternative for reforestation, restoration, and sustainable utilization of both timber and non-timber products. In Mexico and Central America, about 25 pine native species have been used in these plantations [5]. Furthermore, in South America, approximately 16 species have also been introduced in commercial plantations [6].

Plantation success depends on matching species to site conditions, including stressors like drought or poor soil [7]. To improve plantation outcomes under challenging conditions, microbial inoculants, especially ectomycorrhizal fungi (ECM) and beneficial bacteria, have emerged as promising tools to enhance seedling quality and environmental resilience [8].

For instance, inoculation with *Russula roseola* B. Chen, J.F. Liang & X.M. Jiang (Basidiomycota, Russulaceae) and *Suillus luteus* (L.) Roussel improved the establishment of a *Pinus ponderosa* plantation in Patagonian grassland sites with high water stress [9], due to the capacity of ECM fungi to promote plant growth by facilitating the absorption of nutrients and water by the plants and by favoring the release of poorly mobile and low-availability nutrients in the soil, such as phosphorus (P), and protecting the plant from pathogenic organisms [10].

In addition, ECM fungi act synergistically with bacteria, and both microorganisms have been used jointly as inoculants in forest species to improve seedling quality and ensure their establishment in the field [11]. This microbial association offers a sustainable solution to enhance the growth and resilience of forest plantations, reducing the need for chemical fertilizers and improving soil health.

Despite their potential, the role of ECM fungal species [12] and bacteria [13] in promoting the growth and resilience of pine species in the Neotropics remains insufficiently studied. First, the long-term effectiveness of microbial inoculants under diverse Neotropical conditions is poorly understood. Second, most studies focus on single-species inoculants, neglecting potential microbial synergies. Third, the ecological implications of introducing non-native microbial strains remain insufficiently assessed. Finally, mechanistic insights into how inoculants interact with environmental stressors (e.g., competition with native soil microbiota) are limited [12,13].

This review aims to address some of these critical gaps by (i) analyzing current research on *Pinus* species use in the Neotropics and their impact on biodiversity; (ii) identifying the ECM fungi and bacteria used as inoculants in *Pinus* plantations; and (iii) evaluating the synergistic effects of these microorganisms on seedling production and plantation success under Neotropical conditions. By tackling these gaps, the review seeks to advance sustainable forestry practices, improve plantation success, enhance resilience to environmental stressors, and support biodiversity conservation and ecological restoration in the Neotropics.

## 2. Methods

A systematic review was conducted in accordance with the recommendations of the Preferred Reporting Items for Systematic Reviews and Meta-Analysis (PRISMA). Five databases were initially searched (ScienceDirect, Web of Science, Scopus, SciELO, and Redalyc); only four contributed unique records to the final screening stage after deduplication. Additionally, institutional repositories of the Universidad Autónoma Metropolitana (UAM) and Universidad Veracruzana (UV) were accessed. Articles were identified using the keywords ectomycorrhiza, mycorrhizal helper bacteria, *Pinus*, pine, forest plantation, inoculation, inoculant, richness, and diversity, among others, in both English and Spanish.

**Figure 1:**
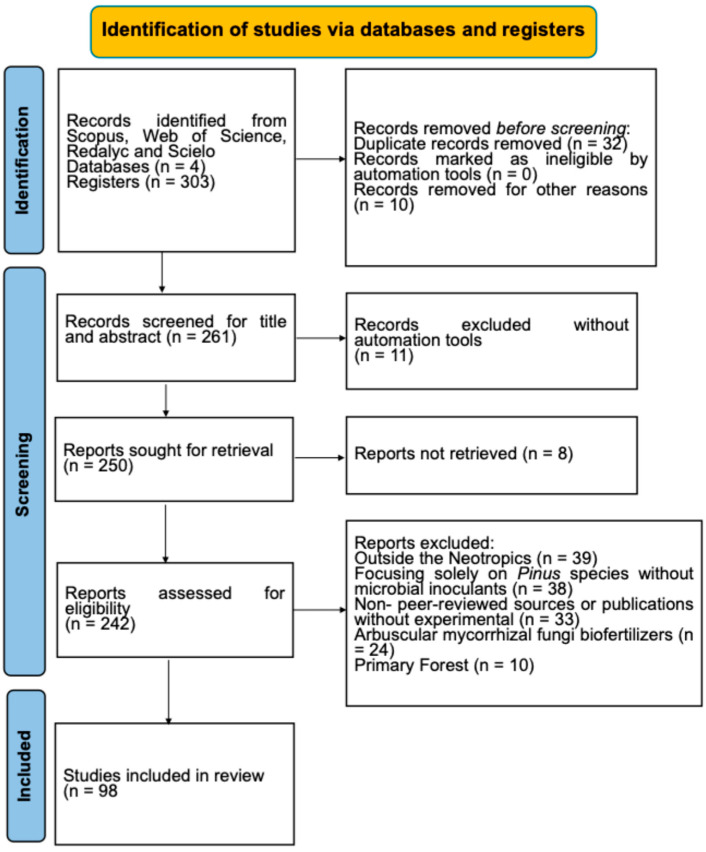
Flow diagram illustrating the methodology of the literature review process following the PRISMA guidelines. The diagram shows the stages of article selection, including identification, screening, eligibility, and inclusion.

The review methodology is delineated in Figure 1, presented in the form of a flowchart. The inclusion criteria for article selection were as follows: (i) articles published between 2001 and 2022, (ii) studies focusing on ECM fungi and/or bacteria in *Pinus* species in the Neotropical region, either native or introduced, (iii) research discussing the effects of microbial inoculants on seedling establishment, growth, or plantation success, and (iv) peer-reviewed publications in English or Spanish.

The exclusion criteria included were (i) studies conducted outside the Neotropics; (ii) articles focusing solely on *Pinus* species without microbial inoculants; (iii) non-peer-reviewed sources (e.g., conference abstracts); (iv) studies without experimental data; (v) arbuscular mycorrhizal fungi biofertilizers; and (vi) primary forests.

The rationale for selecting these keywords was to encompass the most relevant terms related to ECM fungi and bacteria, considering both biological aspects (e.g., fungi, bacteria, inoculation) and the broader ecological and geographical context (e.g., pine species, plantations, diversity). These terms were chosen to reflect both foundational studies on microbial interactions in forests and more recent research that investigates the application of these microbes in plantation systems, particularly in the Neotropics.

A total of 30 articles documenting the use of pine species in forest plantations in the Neotropical region, expanding from Mexico to South America, were reviewed.

Additionally, 40 articles related to the effect of introduced pines in South America on the species richness of native ecosystems were examined.

Furthermore, 28 articles documenting the use of ectomycorrhizal fungi and, more recently, the application of bacteria in different pine species (both native and introduced in the Neotropics) were also analyzed, identifying a list of ectomycorrhizal fungal genera with the potential to enhance the quality of plants intended for plantation purposes. In addition, Global Forest Watch (GFW) updated to 2019, Instituto Nacional de Estadística y Geografía (INEGI) from Mexico and Instituto Nacional de Bosques from Guatemala were consulted for the design of a distribution map of the main localities of commercial forest plantations in the Neotropics.

The information was organized in Excel 2016, and the open-source software QGIS Desktop version 3.28 (Quantum GIS) was used for the geoprocessing of the data and the design of the map.

## 3. Results

### 3.1. Distribution of Pine Species Used in Forest Plantations in the Neotropical Region

The Neotropical zone is considered the tropical zone with the highest biodiversity compared to the Afro-tropical and Southeast Asian zones. However, the accelerated loss of native biodiversity has led to the introduction of forest plantations to conserve, manage, and restore forest ecosystems, as well as to ensure the sustainable provision of timber products and other environmental services [14].

**Figure 2:**
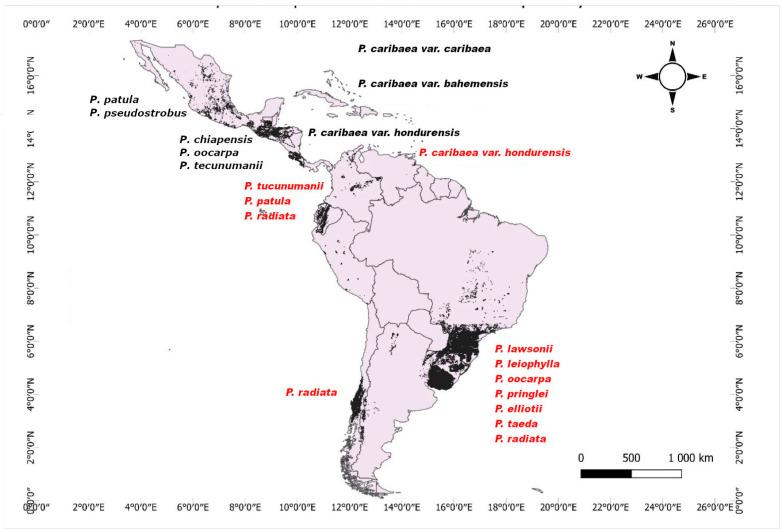
Distribution of pure and mixed *Pinus* Forest plantations in the Neotropics. Species in black letters represent plantations established with native pine species according to their natural geographic range, while red letters indicate plantations with introduced species used mainly for commercial forestry in South America. Classification is based on their natural geographic distribution (see Table S1), as well as official sources used for map design: Global Forest Watch (2019), Instituto Nacional de Estadística y Geografía (INEGI, Mexico), and Instituto Nacional de Bosques (Guatemala).

Currently, forest plantations represent 45% (131 million hectares) of the total global planted forest area, with South America being the region with the highest proportion of planted forests, with 12 million hectares of fast-growing exotic conifers, where the genus *Pinus* occupies approximately 20% of this total (Figure 2).

In Mexico, 51 species of pines have been recorded [1], of which 23 are endemic, 17 species have a distribution range up to the United States of America, and 11 species are distributed from Central Mexico to Nicaragua. Some species such as *P. caribaea* var. *hondurensis* (Sénécl.) W.H. Barrett & Golfari, *P. oocarpa* Schiede ex Schltdl, and *P. tropicalis* Morelet were used in the early forest plantations, mainly for cellulose production (CONAFOR, 2009). Since the 1990s, various programs have been promoted for the establishment of forest plantations with native pines [15] such as *P. patula* Schiede ex Schltdl. & Cham., *P. teocote* Schiede ex Schltdl. & Cham, *P. ayacahuite* var. *veitchii* (Roezl) Shaw, and *P. cembroides* Zucc. exploited as Christmas trees. *Pinus chiapensis* (Martínez) Andresen has been established in high-altitude cloud forest sites due to its rapid growth, lightness, and wood color [16]. *Pinus pseudostrobus* Lindl. is another valuable native species due to the good quality of its wood with a wide altitudinal distribution [17]. *Pinus oocarpa* Schiede ex Schltdl. has been used for carbon sequestration and for timber purposes, and *Pinus greggii* Engelm. ex Parl. has been used for soil restoration purposes, for instance in Atécuaro, Michoacán [18].

On the other hand, species like *Pinus maximinoi* H.E. Moore and *Pinus tecunumanii* Equiluz & J.P. Perry have been underutilized for plantation purposes; however, they present great productive potential due to the low percentage of late wood, which improves the quality of wood and derived products (Table S1).

In Central America and the Antilles, pine diversity decreases significantly to eleven species located from Guatemala to Nicaragua, three species distributed in Cuba, Haiti, and the Dominican Republic (*Pinus cubensis* Griseb., *Pinus occidentalis* Sw., and *Pinus tropicalis*), and two dominant varieties of the “rock pine” forest, a unique and geographically restricted ecosystem: *Pinus caribaea* var. *caribaea* on the Isle of Youth, Cuba, and *P. caribaea* var. *bahamensis* (Griseb.) W.H. Barrett & Golfari in the Bahamas and Turks and Caicos Islands [19]. In El Salvador, Guatemala, Honduras, and Nicaragua, pine plantations have been used to decrease timber demand in natural forests, with species like *P. caribaea* var. *hondurensis* (Sénécl.) W.H. Barrett & Golfari, *P. maximinoi* H.E. Moore, *P. oocarpa* Schiede ex Schltdl and *P. tecunumanii* Equiluz & J.P. Perry being the most utilized [20].

Likewise, in South America, the genus *Pinus* is not naturally distributed; its presence there is the result of introductions for forest plantations, followed in some cases by expansion into surrounding areas [21]. This contrasts with Central America and parts of the Antilles, where some *Pinus* species are native. Currently, 12 million hectares have been designated as planted forests with fast-growing pine and eucalyptus species [22], and approximately 16 Neotropical pine species have been used in forest plantations (Table S2).

In South American countries, many pine species have been utilized for planting and forestry purposes such as *P. caribaea* in Colombia and Venezuela [23]; *P. oocarpa*, *P. patula*, and *P. radiata* in Ecuador; *P. elliottii* Engelm. and *P. taeda* L., in Paraguay and Uruguay; and *P. patula* and *P. radiata* in Peru [24]. Brazil has the highest number of pine species used in forest plantations (12 species) including *P. caribaea*, *P. chiapensis*, *P. elliottii*, *P. kesiya* Royle ex Gordon, *P. leiophylla*, *P. maximinoi*, *P. oocarpa*, *P. patula*, *P. pseudostrobus*, *P. radiata* D. Don, *P. serotina*, and *P. tecunumanii* [25].

Further south in the continent, at Argentina, the first plantations with *P. ponderosa* Douglas ex. C. Lawson date back to 1927 [6]; so far, there are at least 20,000 hectares of planted forests with *P. elliottii*, *P. taeda*, and *P. radiata*, which are mostly cultivated in the central region of this country. *Pinus contorta* Douglas ex Loudon and *P. ponderosa* have been planted in northeastern Patagonia [26], and to a lesser extent also *P. caribaea*, *P. halepensis* Mill., *P. patula*, *P. pinaster* Aiton, and *P. pinea* [25]. In Chile, the most-used species in forest plantations is *P. radiata* [27].

These plantations have reduced the areas of native forest of *Nothofagus* Blume (Nothophagaceae), *Acacia caven* Molina (Fabaceae), *Quillaja saponaria* Molina (Quilaiaceae), and *Maytenus boaria* Molina (Celastraceae) by up to 65% [28] with negative effects on biodiversity. Bravo-Monasterio et al. recorded a decrease in the species richness of native plants in the Aysén region, Chile; species such as *Mulinum spinosum* (Cav.) Pers (Apiaceae), *Leucheria candidissima* Gillies & D. Don (Asteraceae) and *Chloraea alpina* Poepp. (Orchidaceae) have disappeared in areas with a high density of *P. contorta* [29]. Understanding where and which *Pinus* species are planted is key to selecting compatible microbial inoculants. Native species often benefit from local ECM fungi and bacteria, while introduced pines may require co-inoculation with suitable microorganisms. This distribution context supports site-specific inoculation strategies, further discussed in the next sections.

### 3.2. Effect of Pine Species Introduction on Native Ecosystems in South America

The introduction of exotic *Pinus* species in South American ecosystems has led to multidimensional ecological impacts, affecting both aboveground and belowground biodiversity. These impacts are observed across various taxonomic groups, including plants, animals, and microorganisms. Species richness of plant species decreases by 28% to 65% in introduced forest plantations compared to grasslands, steppes, paramo, and temperate forests dominated by diverse species of *Nothofagus* in the Patagonia region [30,31,32]. A similar trend has been observed in fauna: although some studies report comparable numbers of generalist species (e.g., birds and insects) between pine plantations and native forests, specialist species consistently decline (Table S3).

For instance, Hernandes-Volpato et al. demonstrated that the number of generalist bird species found in *Araucaria angustifolia* (Bertol.) Kuntze (Araucariaceae) forests (9 species) was statistically similar to the number of species (11 spp.) recorded in *P. elliottii* forest plantations [33]; however, the number of specialist birds decreased (8 and 3 species, respectively). Rubio et al. did not report differences in mammal species richness between deciduous temperate forest (7 species) and mature (5 species) and young *P. radiata* plantations (6 species) [34]; Renner et al. recorded a higher species richness of *Odonata* spp. (Artropoda, Insecta) in *P. elliottii* plantations (27 species) than in a pristine *Araucaria* Forest in Brazil (22 species) [35] (Table S3).

While forest plantations can act as temporary reservoirs of biodiversity, most species are generalists, indicating their partial influence on community assemblages [33]. Similarly, the soil microorganism composition has also been affected by the introduction and invasion of exotic pines, altering plant composition and, therefore, modifying the percentage and chemical composition of the organic matter, as well as the exudates released into the soil, directly affecting communities of microorganisms that play a key role in nutrient cycling [36,37,38].

For example, in the highlands of Northern Andes, the richness of ectomycorrhizal fungi (ECM) in introduced pines is significantly lower than in natural habitats, with at least three predominant species: *Rizhopogon vulgaris* (Vittad.) M. Lange, *Suillus luteus* (L.) Roussel, and *Thelephora terrestris* Ehrh. ex Fr. [39]. Policelli et al. compared ECM fungi richness in *P. contorta*, recording five fungal species (*Suillus luteus*, *Hebeloma* sp., *Thelephora terrestris*, *Sistotrema* sp., and *Amanita muscaria*), while eight species were recorded in *Nothofagus antarctica* native forests (*Cortinarius* sp., *Clavulina* sp., *Inocybe* sp., *Tomentella* sp., *Tricholoma* sp., *Porpoloma terreum*, *Ricknella minuta*, and *Sistotrema* sp.) [37].

Several ECM fungal species have been co-introduced with their hosts through forest soil [40] and, in commercial products, used as substrates [41]. Gundale et al. mention that native ECM fungi species do not form associations with non-native *Pinus* due to the lack of long dispersal mechanisms or non-persisting spores in the soil for large periods of time, making it difficult for non-native species to establish successfully [42].

Microbial inoculants—particularly those involving native ECM fungi—are often promoted as ecologically beneficial alternatives in forestry practices. To date, there are no studies that evaluate whether their inoculation can mitigate the ecological impacts of exotic pines on native soil microbiota. This represents a significant knowledge gap, especially in restoration contexts where microbial inoculants could potentially support the reestablishment of native soil communities.

**Figure 3:**
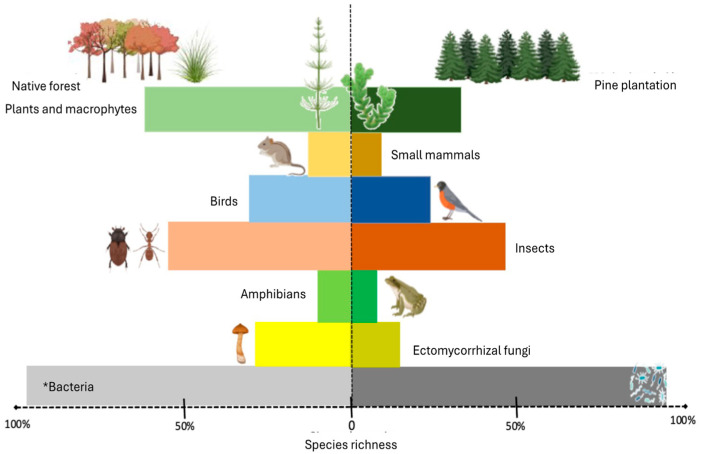
Comparative species richness (in %) across major taxonomic groups between native forests (**left**) and *Pinus* plantations in South America (**right**). The values represent general trends reported in the literature and are intended for visual comparison only. For most groups, synthesis is based on multiple references (see Table S3), except for bacteria (*), which is based on a single study [43]. This figure does not display variance due to heterogeneity in sampling methods and scales across sources (source: author’s own work).

Regarding bacterial communities, Almonacid-Muñoz et al. reported that the soil organic layer composition affects the bacterial species richness, finding 444 amplicon sequence variants (ASVs) in a *P. radiata* plantation compared to 534 ASVs in a native *Nothofagus obliqua* (Mirb.) Oerst forest [43]. However, changes in bacterial diversity have not been sufficiently explored in these scenarios, making their analysis crucial for soil microbial diversity conservation (Figure 3).

In summary, while pine plantations can support certain biodiversity components, their introduction often leads to a simplification of both aboveground and belowground communities. These changes highlight the importance of evaluating microbial inoculants not only for their benefits in plantation success but also for their potential to conserve or restore microbial diversity.

### 3.3. Importance of Mycorrhization and the Use of Bacteria in Pine Seedling Production

Mycorrhizal fungi play a crucial role in the establishment of pine forest plantations [44], providing a specific habitat for diverse bacterial communities that in turn promote plant growth and environmental resistance [44], thereby increasing plantation survival in the field [45].

Specifically, pines form symbiotic associations with ECM, which facilitate the plant’s water and nutrient absorption area through extraradical mycelium formation in the soil, hence enhancing plant nutritional quality, disease resistance, and drought stress tolerance.

Approximately 90 ECM species can synthesize proteins (aquaporins) that regulate water acquisition and transport from the fungus to the host plant by controlling the permeability of the hyphal cell membranes [46]. The symbiotic water absorption efficiency by the host enhances defense against diseases; for instance, Chu et al. reported that inoculating *Pinus tabulaeformis* Carr with *Suillus laricinus* (Berk.) O. Kuntze increased plant water content, correlating with seedling mortality caused by the nematode *Bursaphelenchus xylophilus* Steiner & Buhrer [47].

These fungal communities are an important resource for other soil organisms, especially in the rhizosphere, where they affect bacterial community composition, for example, plant growth-promoting rhizobacteria (PGPR), plant growth-promoting bacteria (PGPB) and mycorrhizal helper bacteria (MHB). The latter group of bacteria is the most crucial for the establishment of ectomycorrhizal fungi. MHB can stimulate the metabolism of both the host plant and ectomycorrhizal fungi through different mechanisms, such as (i) degrading complex and toxic soil compounds like polyphenols, (ii) promoting host root development and formation of secondary roots, (iii) increasing carbon assimilation, (iv) boosting auxin production to stimulate pre-symbiotic mycelial growth, (v) regulating gene expression involved in mycelial branching, and (vi) regulating hormones like indoleacetic acid and gibberellins involved in mycorrhizal formation [48].

The suppression of pathogenic fungi is one of the main beneficial features of MHB. For instance, *Lysobacter* sp. SB-K88 releases antifungal compounds like xanthobaccins that hinder mycelial development, or through some enzymes like chitinases or glucanases [49]. However, the exudation of different antifungal compounds can also inhibit the growth of ectomycorrhizal fungi. Some species are more tolerant than others; for example, *Streptomyces* sp. AcH 505 promotes the growth of *Amanita muscaria* and *Suillus bovinus* (L.) Roussel but limits the growth of *Hebeloma cylindrosporum* Romagn, due to its susceptibility to antibiotic components [50].

So far, it has been found that ECM fungi interact positively, neutrally, or negatively with many MHB strains belonging to various groups, especially Firmicutes, Gram-positive Actinobacteria, and Gram-negative Proteobacteria [51]. However, the difficulty in culturing them has limited research to determine if other bacterial groups such as Acidobacteria may have high potential as forest inoculants alongside symbiotic fungi to optimize the formulation of biofertilizers based on ECM fungi.

To date, the interaction between ECM fungi and bacteria is not a common practice in pine species inoculation in the Neotropical forestry region [13]. Recent research has identified certain bacterial strains that play a crucial role in promoting ectomycorrhizal symbiosis. These findings demonstrate that integrating these bacteria alongside ECM fungal inoculants results in a synergistic effect, notably enhancing seedling survival rates in both controlled greenhouse environments and field conditions [52,53]. In greenhouse studies, parameters such as seedling height, root and shoot biomass, and fungal colonization rates were measured to assess growth and inoculation effectiveness, and in field studies, survival rates, growth performance, and persistence of ECM colonization were measured to evaluate the adaptation and success of inoculated seedlings in plantation environments (see Table S4 for detailed references and methodologies).

This combined approach not only bolsters seedling survival but also fosters greater adaptability, tolerance to environmental stressors, and improved physio-morphological development. Such advancements are pivotal for ensuring the successful establishment of future forest plantations, promising a more resilient and thriving ecosystem.

**Figure 4:**
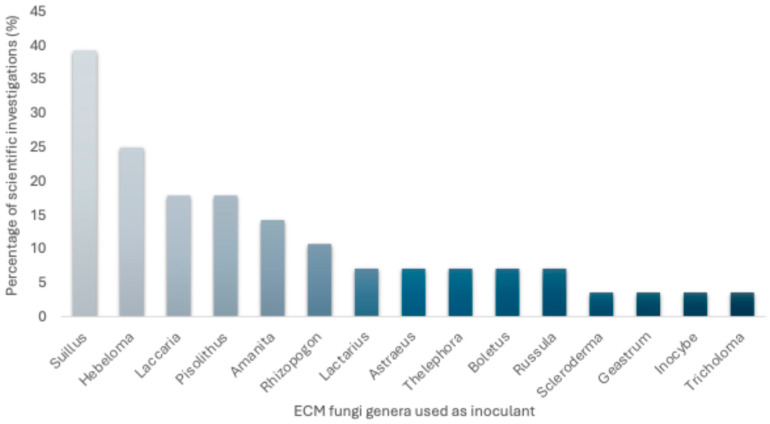
Percentage of analyzed scientific investigations reporting the use of ectomycorrhizal (ECM) fungi as inoculants in Neotropical pines (source: author’s own work).

Based on the diversity of ECM fungi examined in the literature, the most frequently used genera in pine inoculation studies across the Neotropics are highlighted below. An analysis of the reviewed articles shows that 39% of the studies employed fungal species of the genus *Suillus* as a source of mycorrhizal inoculation in seedlings of *P. maximinoi*, *P. greggii*, *P. patula*, *P. hartwegii*, *P. tecunumanii*, *P. pseudostrobus*, and *P. oocarpa*, as well as in introduced species like *P. ponderosa* and *P. radiata*. Twenty-five percent of the studies reported the use of *Hebeloma* species as mycorrhizal inoculants in the roots of *P. ayacahuite*, *P. greggii*, *P. montezumae*, *P. pringlei*, *P. ponderosa*, *P. patula*, and *P. pseudostrobus*. Seventeen percent of the investigations reported the use of species of *Pisolithus* Alb. & Schwein *Laccaria* Berk. & Broome and *Amanita* Pers. in pines with wider distribution ranges such as *P. pseudostrobus*, *P. montezumae*, and *P. greggii*. Other ECM fungi genera like *Rhizopogon* Fr., *Lactarius* Pers., *Astraeus* Morgan, *Thelephora* Ehrh. ex Willd., *Boletus* L., *Russula* Pers., *Scleroderma* Pers., *Geastrum* Pers., *Inocybe* and *Tricholoma* have been used in low proportions and with fewer host pine species (Figure 4).

This classification reflects their relative prevalence in published trials and helps identify which genera have shown the most practical relevance across pine species.

*Amanita*, *Boletus*, *Suillus*, and *Rhizopogon* species are reported more frequently in South American countries as forest inoculants for pines. Mexico has the highest number of reports on the use of ECM, with at least 17 scientific reports evaluating seedling quality of pines using different ECM fungi, followed by Chile (3), Colombia (3) and Argentina (2).

In parallel, studies on bacterial inoculants have identified key genera used to enhance pine seedling growth and quality. Regarding the use of bacteria, 20% of the analyzed studies registered at least 12 genera of bacteria used as promoters of pine seedlings in relation to the increase in growth and quality in the Neotropics [54,55]. Many of these bacteria function as PGPB or PGPR, as well as mycorrhizal helper bacteria. The most frequently used bacterial genera in these studies are *Bacillus*, *Cohnella*, and *Pseudomonas* (see Table S4).

These genera have shown diverse effects [56] on pine seedlings depending on their bacterial group (PGPB, PGPR, or MHB) and their interaction with specific pine species. The pine species used in bioassays include *P. chiapensis***,** *P. cembroides***,** *P. montezumae*, and *P. pseudostrobus* in Mexico, and *P. taeda* in Brazil.

*Bacillus* species are widely recognized for their ability to promote plant growth through several mechanisms such as nitrogen fixation, the production of phytohormones (e.g., auxins), and improving nutrient uptake, particularly phosphorus [57]. These bacteria also help suppress soil-borne pathogens, contributing to plant health. In Neotropical pine species, *Bacillus* spp. have been associated with growth-promoting mechanisms, such as auxin production, phosphate solubilization, and siderophore production, making them key players in enhancing the establishment of healthy pine forests [58].

Moreover, *Cohnella* spp. significantly enhance the colonization of mycorrhizal fungi, which are critical for the growth and survival of pine seedlings. In some studies, inoculation with *Cohnella* has led to substantial increases in seedling dry weight and root development, particularly when co-inoculated with ectomycorrhizal fungi [13].

*Pseudomonas* species are primarily known as PGPR but also act as MHB, promoting plant growth by enhancing nutrient availability, producing plant growth regulators, and suppressing pathogens through antimicrobial compounds [59]. However, the effects of *Pseudomonas* spp. on pine seedlings can vary. For example, inoculation with *Pseudomonas fluorescens* in *Pinus taeda* led to decreased growth under certain conditions [60]. Despite this, *Pseudomonas* spp. is often beneficial when combined with mycorrhizal fungi, leading to improved seedling performance and establishment.

The use of different bacterial genera as bioinoculants holds significant potential for enhancing pine seedling growth in the Neotropics. The synergistic effects of these microorganisms can boost seedling survival and growth, supporting sustainable forestry practices [13,53]. However, further research is needed to assess long-term impacts and optimize inoculation strategies for different pine species under varying environmental conditions.

### 3.4. Response of Pine Seedlings to Mycorrhizal Inoculation

**Figure 5:**
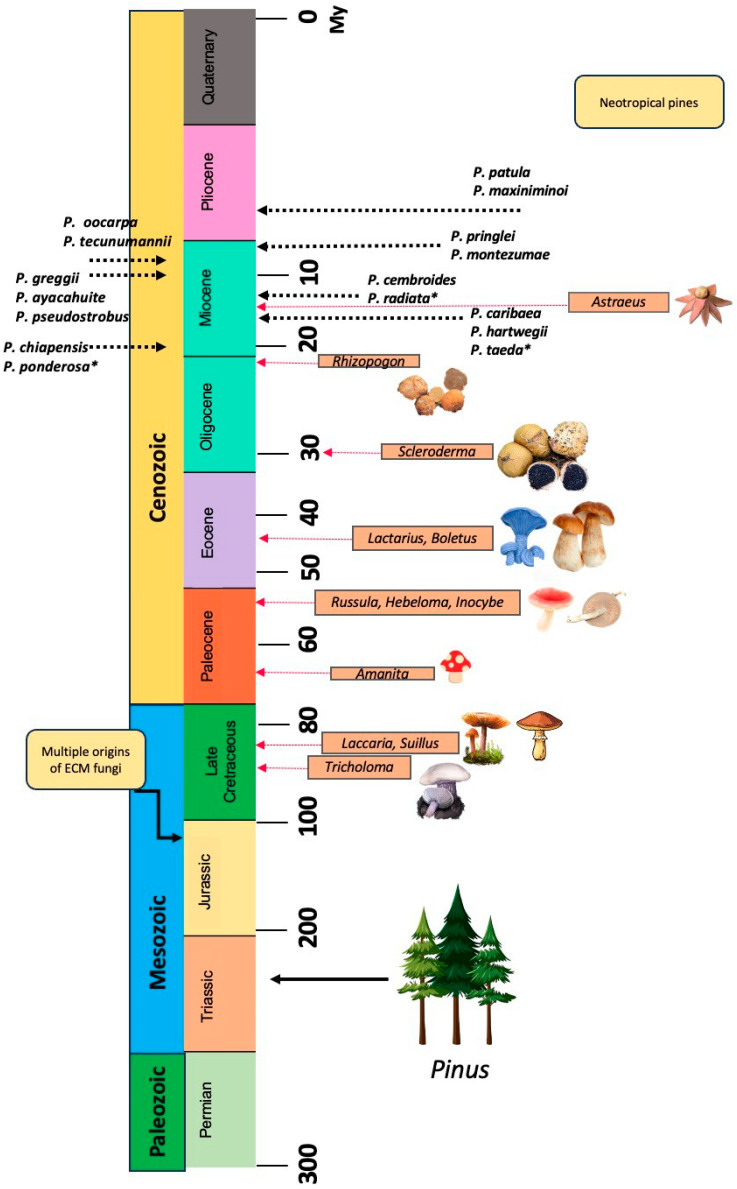
Evolutionary timeline (in millions of years, My) showing the divergence of selected Neotropical *Pinus* species (**left**) and major ectomycorrhizal (ECM) fungal genera used in inoculation studies (**right**). Arrows indicate estimated divergence periods based on phylogenetic evidence. Introduced pine species are marked with an asterisk. This evolutionary context illustrates the asynchronous origin of fungal symbionts and their hosts (source: author’s own work).

The benefits offered by microorganisms, mainly ECM fungi, depend primarily on the fungal species used, the substrate, and the host plant species. Among the ECM fungal genera mentioned above, *Suillus* and *Hebeloma* have shown positive effects on plant quality, particularly by enhancing growth and development when used as inoculants in Neotropical pine seedlings [61,62,63]. However, there is not a clear trend regarding the evolutionary relationship between ECM fungi reported as inoculants and Neotropical pine species (Figure 5).

Hence, this may be because most of the assays are conducted in greenhouses, with quite a few in the field, and possibly that the selection of mycorrhizal fungi depends on the local environmental conditions and the ecological factors. Mexican and Central American native pine species exhibit early divergence compared to the repeated evolution of different ECM lineages such as *Suillus* and *Hebeloma*, which have repeatedly evolved since the Cretaceous, while some other ECM fungal genera have lost gene sets that had allowed associations with numerous plant genera [64].

Nonetheless, fungal genera like *Suillus* specifically encode novel genes involved in mycorrhizal formation with a wide range of host species within the Pinaceae family, exhibiting a high capacity for colonization with native pines and acting as pioneer species to colonize non-native pine seedlings [36]. In the case of *Suillus* species and their interaction with different Neotropical pine species, some assays report that the inoculation with *Suillus caerulescens* A.H.Sm. & Thiers promoted a significant increase in growth in *P. greggii* seedlings: up to 30% in stem height, 28% in basal diameter, 57% in aboveground biomass, and 68% in root biomass compared to the control [61]. However, in *P. hartwegii* seedlings inoculated with *Suillus brevipes* (Peck) Kuntze, no significant differences in growth were reported, even with 60% fungal colonization [65].

Nevertheless, in *P. pseudostrobus*, a significant increase of 28% in height, 30% in basal diameter, and ca. 40% in the total content of total polyphenols was observed in seedlings inoculated with *Suillus decipiens* (Peck) Kuntze compared to the non-inoculated ones [66]. Although, when *S. decipiens* is co-inoculated with *Amanita stranella* E.-J. Gilbert & Snell in *P. pseudostrobus* seedlings, increases of up to 59% in stem height and 50% in total polyphenols compared to the control were reported. This synergistic effect has also been reported in *P. tecunumanii* seedlings inoculated with *Suillus luteus* and *Amanita muscaria*; the seedlings recorded a 9% increase in height when only inoculated with *S. luteus*, and 27% when they were inoculated with both fungi; in addition, dry weight increased by 29%.

These authors also reported increases of up to 40% in dry biomass in *P. oocarpa* seedlings and 0.19% in phosphorus (P) in *P. patula* seedlings when *S. luteus* was inoculated jointly with *A. muscaria*. Other authors reported increases of 17% and 10% in height and 14% and 20% in dry weight in *P. patula* and *P. maximinoi*, respectively, inoculated with *S. luteus* compared to controls, showing fungal colonization percentages below 30% in both pine species [67].

In Argentina, *P. ponderosa* seedlings inoculated with *S. luteus* showed no significant differences in growth except in stem height, with an 8% increase compared to the control, presenting between 38% and 70% of mycorrhizal colonization [68]. Assays with *Hebeloma* showed a similar trend. Artega-León et al. inoculated *P. ayacahuite* with *Hebeloma mesophaeum* (Pers.) Quél., obtaining an increase in dry weight of 63%, height of 20%, nitrogen (N) of 100%, and P of 63% compared to non-inoculated seedlings, as well as reporting root colonization percentages between 41% and 59% [63]. Rentería-Chávez et al. inoculated *Hebeloma leucosarx* P.D. Orton in *P. greggii* seedlings, observing increases in dry biomass of 800%, stem height of 77%, and stem diameter of 71% compared to the control [69]. Another assay performed by López-Gutiérrez et al. with *Hebeloma alpinum* (J. Favre) Bruchet, associated with *P. pringlei*, reported considerable increases in biomass (437%), stem height (44%), stem diameter (83%), and N and P concentrations in the seedling tissue (100%) [45]. It is also relevant to mention that the *Hebeloma* genus has a wide distribution range; thus, it has been reported to be associated with multiple hosts in temperate forests of Mexico [70].

However, this genus has a wide distribution range, so it has also been used in Argentina, associated with *P. ponderosa*, but no significant increases in stem height and diameter were reported when seedlings were inoculated, compared to non-inoculated ones, even when root fungal colonization percentages ranged from 11% to 46% [62,68]. These findings suggest that the greatest benefits are observed when ECM fungi that are used as inoculants come from the same native distribution areas of the hosts, even when the pine species occur outside their natural range. However, some species like *P. ponderosa* that have been introduced into Neotropical areas may not respond efficiently to different ECM species, especially those that maintain an obligatory relationship with pines, such as *Suillus*. Likewise, the bacteria used in bioassays with pines so far come from commercial strains or are isolated from the rhizosphere of other hosts or ecosystems with different characteristics from the site where pine seedlings are expected to establish [52].

According to the information and data given by the analyzed articles, only 20% of the assays made use of bacteria as inoculants in pine seedlings, with Mexico and Brazil being the countries with the highest number of studies. The most examined bacterial genera have been *Bacillus*, *Cohnella*, and *Pseudomonas*. Domínguez-Castillo et al. inoculated *P. chiapensis* seedlings with bacterial strains and obtained higher germination rates when inoculated with *Bacillus*, *Dyella* Xie and Yokota, and *Paraburkholderia* Sawana [54]. In *P. cembroides* inoculated with *Cohnella* sp., an 8% increase in height and 10% increase in basal diameter were reported compared to the control; in addition, when it was co-inoculated with *Laccaria proxima* (Bound.) Pat., height increased by 66% and stem diameter by 52% compared to non-inoculated seedlings [55].

Further, in *P. montezumae* seedlings, dry weight increased by 45% with *Cohnella* sp. and basal diameter increased by 125%; in addition, when seedlings of the same pine species were inoculated with *Azospirillum*, the dry weight increased by 91%, and basal diameter by 150%, compared to non-inoculated plants. At the same time, when these pine seedlings were inoculated with *H. mesophaeum* jointly with *Cohnella* sp., dry weight increased by up to 325%, this value being 472% with *Azospirillum* [14].

In Neotropical areas, where pine species such as *P. taeda* have been introduced, inoculation with *Bacillus subtillis* increased biomass in seedlings, while *Pseudomonas fluorescens* showed negative effects on seedling growth [60]. These findings highlight the recommendation to use both ECM and bacteria jointly as bioinoculants in pine plantation establishment.

The synergistic interaction between these groups of symbiotic microorganisms, fungi, and bacteria promotes optimal development and improves the growth and quality of pine seedlings. Although there are plenty of areas of research to explore, the overall results support the importance of making the most of the synergistic association between ECM fungi and bacteria to improve the productivity and sustainability of both native and introduced pine forest plantations. Additionally, this strategy could be crucial for advancing towards more efficient and environmentally respectful forest management in the Neotropical context.

### 3.5. Cost and Composition of Commercial Microbial Inoculants in Mexico

In Mexico, the use of native ECM fungi represents a promising strategy to improve the quality and production of pine seedlings in Neotropical forestry. However, these native species are still rarely included in the commercial inoculants used in forest nurseries, where *Pisolithus tinctorius* remains the most frequently applied ectomycorrhizal species. This section presents a detailed case study on the costs associated with producing native ECM inoculants in the Mexican context, based on recent field experiences and published reports. It also includes a comparative overview of commercial microbial products available in Mexico, highlighting their composition and cost (Table S5). Some studies have demonstrated the efficiency of native ECM. For instance, Valdés-Ramírez et al. reported a 12% increase in biomass in *P. pseudostrobus* seedlings 6 months after inoculation with *Scleroderma texense* Berk., whereas seedlings inoculated with a commercial strain of *Pisolithus tinctorius* showed an 8% decrease in biomass compared to the control [71].

However, after 12 months of inoculation, a 6% decrease was observed with *S. texense* and a 7% increase with commercial *P. tinctorius*. Recently, Baeza-Guzmán et al. (unpublished, personal communication) reported a 16% increase in stem height in *P. patula* seedlings inoculated with *Laccaria/Suillus* compared to those inoculated with the commercial product. In terms of costs, Carrasco-Hernández et al. considered that a native mycorrhizal inoculant (powder) may have an approximate cost of USD 99.61 per kilogram [12]. While some commercial products available on the market are significantly more expensive, others can be produced through conventional methods at a lower cost. For instance, Baeza-Guzmán et al. developed a native inoculant for *P. pseudostrobus* at an approximate cost of USD 0.18 per gram, which proved to be effective in enhancing pine seedling quality [66]. This inoculum, unlike that reported by Carrasco-Hernández et al., began with obtaining fruiting bodies in the field, which increased the costs of collection and transportation. Fungi recollections were made twice a week for two months with the intervention of two people per day (USD 4.72 per person), costing USD 118 for the eight visits in gasoline. For drying the fungi, a homemade wooden dehydrator with a capacity of 2.5 kg was made, costing USD 29.5. The dehydration required a manual mill costing USD 3.54 per kilogram.

In storage, the cost of electricity every two months represented USD 8.86. In total, 10 kg of fresh mushrooms was collected, which was converted into dry and pulverized inoculum, resulting in 2 kg. It is important to standardize these processes to increase the efficiency of native inoculant production and transfer the technology to local people for introducing this new practice in the communal forest nurseries to minimize costs in products made from microorganisms. This could be critical to contribute both sustainable use of resources linked to pine production and local mushroom production in terms of achieving food sovereignty.

## 4. Conclusions

This study presents a novel approach to pine seedling inoculation in the Neotropical region by focusing on native ectomycorrhizal (ECM) fungi and bacteria, and by explicitly addressing four key knowledge gaps.

First, while the long-terms effects of microbial inoculation remain underexplored, this analysis of early-stage growth responses provides a foundation for predicting potential long-term benefits. By highlighting studies that show sustained improvements in biomass, nutrient uptake, and stress tolerance during the initial establishment phase, we suggest that microbial inoculation holds promise for lasting plantation success—though further longitudinal studies are clearly needed.

Second, although there is significant research on the use of ECM fungi in other regions, few studies have explored the interaction between native fungi and bacteria in Neotropical pine species, especially considering the region’s unique ecological conditions. This review goes beyond traditional inoculation methods that commonly rely on non-native or commercial strains (e.g., *Pisolithus tinctorius*), instead emphasizing native species, such as *Suillus* and *Hebeloma*, and the role of beneficial bacteria like *Bacillus* and *Pseudomonas*. Their demonstrated synergistic effects on seedling biomass and nutrient uptake directly address the need of optimizing combinations of ECM fungi and bacteria rather than relying on single-species inoculants.

Third, this review highlights the ecological risks of non-native inoculants and underscores the benefits of using native microbial strains that are better adapted to local pine hosts and environmental conditions. By promoting native ECM fungi and rhizobacteria, the review supports microbiota-based strategies that are ecologically sound and aligned with biodiversity conservation goals.

Fourth, this combination of fungi and bacteria has been shown to enhance the growth and biomass of various Neotropical pine species, such as *Pinus maximinoi*, *Pinus greggii*, and *Pinus oocarpa*, which are vital for regional forest restoration efforts. The synergistic relationship between ECM fungi and bacteria is crucial for the establishment of pine plantations. ECM fungi enhance nutrient absorption and plant growth, while rhizobacteria promote plant defense mechanisms and resilience against pathogens.

Despite the advances made, key challenges persist in the Neotropics, including limited research on microbial diversity, host specificity, and the availability of scalable, high-quality inoculants. This study has several limitations that should be considered. Firstly, inoculation effectiveness varied across different pine species, which may be influenced by environmental factors and soil characteristics not fully accounted for in this study. Moreover, most existing studies lack long-term field assessments, underlining the need for future research on the sustainability of inoculation practices and their broader ecological implications.

In summary, the tripartite symbiosis between ECM fungi, beneficial bacteria, and pine species represents a promising strategy to enhance the biodiversity, resilience, and productivity of forest plantations in the Neotropics.

This review highlights how such microbial partnerships contribute not only to soil conservation and agrosilvopastoral system improvement but also to the ecological restoration of degraded landscapes. By promoting the use of native microbiota, these approaches support biodiversity conservation and reduce the ecological risks posed by non-native inoculants. Beyond environmental benefits, this strategy holds significant social and economic value: sustainable forestry practices that integrate microbial inoculation can generate livelihood opportunities, stabilize incomes from forest products, and foster long-term environmental stewardship among local communities.

## Supplementary Materials

The following supporting information can be downloaded at https://www.mdpi.com/article/10.3390/jof11050393/s1, Table S1: Natural distribution of *Pinus* species in the Neotropics and the use of these species in forest plantations in South America; Table S2: Native species of *Pinus* that have been used in forest plantations in Mexico and Central America and introduced in South America; Table S3: Species richness in different taxonomic groups in pine plantations in the neotropical region of South America compared to the natural habitat present; Table S4: Ectomycorrhizal fungi and bacteria with potential use as inoculants to enhance the physio-morphological development of neotropical pine species of interest for use in forest plantations; Table S5: Inoculants based on mycorrhizae and bacteria suggested and used in forest plant production that are marketed in Mexico. Refs. [72,73,74,75,76,77,78,79,80,81,82,83,84,85,86,87,88,89,90,91,92,93,94,95,96,97,98,99,100,101,102,103,104,105,106,107] are cited in the Supplementary Materials.

## Abbreviations

ECM	ectomycorrhizal fungi
PGPR	plant growth-promoting rhizobacteria
PGPB	plant growth-promoting bacteria
MHB	mycorrhizal helper bacteria
