# Supplementary material for: Pine Forest Plantations in the Neotropics: Challenges and Potential Use of Ectomycorrhizal Fungi and Bacteria as Inoculants
Source: J Fungi (Basel). 2025 May 20;11(5):393. doi: 10.3390/jof11050393 (PMC12113481; doi:10.3390/jof11050393)
Supplement: Supplementary file 1 [file jof-11-00393-s001.zip › jof-3611902-supplementary.pdf]

**Table S1.** Natural distribution of *Pinus* species in the Neotropics and the use of these species in forest plantations in South America.

| Specie | Distribution | Region | FP South America |
|---|---|---|---|
| *P. ayacahuite* Ehrenb. ex Schltdl. var. *ayacahuite* | Mexico: Veracruz, Puebla, Hidalgo, Tlaxcala, Guerrero, Oaxaca, Chiapas; Guatemala; El Salvador; Honduras | Mexico-Central America | FP |
| *P. ayacahuite* Ehrenb. ex Schltdl. var. *veitchii* (Roezl) Shaw | Mexico: Veracruz, Puebla, Hidalgo, Tlaxcala, Guerrero, Oaxaca, Chiapas; Guatemala; El Salvador; Honduras | Mexico-Central America | - |
| *P. caribaea* Morelet var. *hondurensis* (Senecl.) W. H. Barrett et Golfari | Mexico: Quintana Roo; Guatemala; Belize; Honduras; El Salvador; Nicaragua | Mexico-Central America | FP |
| *P. caribaea* var. *caribaea* (Griseb.) W.H.Barret | Cuba, Isla de la Juventud | Antilles | - |
| *P. caribaea* var. *bahamensis* (Griseb.) W.H.Barret | Bahamas; The Turks and Caicos Islands | Antilles | - |
| *P. cembroides* Zucc. subsp. *cembroides* | USA to Mexico: The Sierra Madre Occidental and Sierra Madre Oriental, up to northern Puebla. | USA-Mexico | - |
| *P. cembroides* Zucc. subsp. *orizabensis* D. K. Bailey | USA to Mexico: The Sierra Madre Occidental and Sierra Madre Oriental, up to northern Puebla | USA-Mexico | - |
| *P. chiapensis* (Martínez) Andresen | Mexico: Oaxaca, Chiapas, Veracruz, Guerrero, Oaxaca; Guatemala | Mexico-Central America | FP |

| | | | |
|---|---|---|---|
| *P. cubensis Griseb.* | Cuba | Antilles | - |
| **P. culminicola* Andresen et Beaman | Mexico: Coahuila y Nuevo León | Mexico | - |
| *P. devoniana* Lindl | Mexico: Nayarit, Zacatecas, Jalisco, Colima, Michoacán, Hidalgo, Estado de México, Puebla, Morelos, Guanajuato, Tlaxcala, Guerrero, Oaxaca, Veracruz, Chiapas; Guatemala, | Mexico-Central America | FP |
| **P. discolor* D. K. Bailey et Hawksw | USA: Arizona, New Mexico; Mexico: Sonora, Chihuahua, Durango. | USA-Mexico | - |
| **P. douglasiana* Martínez | Mexico: Durango, Chihuahua, Sonora, Sinaloa, Nayarit, Jalisco, Michoacán, Colima, Estado de México, Guerrero, Oaxaca, Chiapas. | Mexico | - |
| **P. georginae* Perez de la Rosa | Mexico: Southeast of the Ameca River, Jalisco, Northwest Mexico | Mexico | - |
| **P. greggii* Engelm. ex Parl.<br>var. *australis* Donahue et Lopez | Mexico: Coahuila, Nuevo León, San Luis Potosí, Hidalgo | Mexico | - |
| **P. greggii* Engelm. ex Parl. var. *greggii* | Mexico: Coahuila, Nuevo León, San Luis Potosí, Hidalgo | Mexico | - |
| *P. hartwegii* Lindl | Mexico: Chiapas, Chihuahua, Coahuila, Colima, Durango, Guerrero, Hidalgo, Jalisco, Estado de México, Michoacán, Morelos, Nuevo León, Oaxaca, Puebla, Tamaulipas, Tlaxcala y Veracruz; Guatemala: El Quiché, | Mexico-Central America | - |

| | | | |
|---|---|---|---|
| | Guatemala, Huehuetenango, Quezaltenango, Sacatepequez, San Marcos, Sololá,Totonicapán; Honduras: Cerro Santa Bárbara | | |
| **P. herrerae* Martínez | Mexico: Chihuahua, Sinaloa, Durango, Jalisco, Michoacán, Guerrero | Mexico | - |
| *P. jaliscana* Perez de la Rosa | México: Jalisco | Mexico | - |
| **P. jeffreyi* Balf. | USA: Oregon, Nevada, California; México: Baja California | USA-Mexico | - |
| **P. johannis* Rob.-Pass | Mexico: Coahuila, Nuevo León, Zacatecas | Mexico | - |
| **P. lagunae* (Rob.-Pass) Passini | Mexico: Baja California Sur: Sierra de la Laguna, | Mexico | - |
| **P. lambertiana* Douglas | USA: Oregon, Nevada, California; México: Baja California: Sierra de San Pedro Mártir | USA-Mexico | - |
| *P. lawsonii* Roezl ex Gordon | Mexico: Jalisco, Michoacán, Estado de México, Morelos, Puebla, Guerrero, Oaxaca, Hidalgo, Morelos. | Mexico | FP |
| *P. leiophylla* Schiede ex Schltdl. et Cham. | Mexico: Chihuahua, Durango, Zacatecas, Jalisco, Sinaloa, Nayarit, Colima, Michoacán, Estado de México, Tlaxcala, Puebla, Morelos, Veracruz, Oaxaca | Mexico | FP |
| **P. lumholtzii* B. L. Rob et Fernald | Mexico: Aguascalientes, Chihuahua, Durango, Guanajuato, Jalisco, Nayarit, Sinaloa, Sonora, Zacatecas | Mexico | - |
| *P. luzmariae* Perez de la Rosa | Mexico: Durango, Jalisco, Nayarit, Oaxaca, Michoacán | Mexico | - |

| | | | |
|---|---|---|---|
| **P. maximartinezii* Rzed. | Mexico: Zacatecas | Mexico | - |
| *P. maximinoii* H. E. Moore | Mexico: Chiapas, Colima, Durango, Guerrero, Hidalgo, Jalisco, Estado de México, Michoacán, Morelos, Nayarit, Oaxaca, Puebla, Sinaloa, Tlaxcala, Veracruz; Guatemala; Honduras; El Salvador; and Nicaragua. | Mexico-Central America | FP |
| *P. montezumae* Lamb. var. *gordoniana* (Hartw. ex Gordon) Silba | Mexico: Nuevo León, Coahuila, Tamaulipas, Hidalgo, Tlaxcala, Puebla, Veracruz, Estado de México, Morelos, Michoacán, Jalisco, Guerrero, Oaxaca y Chiapas; Guatemala: Huehuetenango, Quiché, San Marcos, Quezaltenango, Totonicapán, Sololá, Chimaltenango, Guatemala and Jalapa | Mexico-Central America | - |
| *P. montezumae* Lamb. var. *montezumae* | Mexico: Nuevo León, Coahuila, Tamaulipas, Hidalgo, Tlaxcala, Puebla, Veracruz, Estado de México, Morelos, Ciudad de México, Michoacán, Jalisco, Guerrero, Oaxaca and Chiapas; Guatemala: Huehuetenango, Quiché, San Marcos, Quezaltenango, Totonicapán, Sololá, Chimaltenango, Guatemala and Jalapa | Mexico-Central America | FP |
| *P. muricata* D. Don | USA: California; Mexico: Baja California | USA-Mexico | - |
| **P. nelsonii* Shaw | México: Nuevo León, San Luis Potosí and Tamaulipas | Mexico | - |
| *P. occidentalis Sw.* | Haiti; Dominican Republic | Antilles | FP |
| *P. oocarpa* Schiede ex Schltdl. | Mexico: Sonora, Sinaloa, Durango, Nayarit, Zacatecas, Jalisco, Michoacán, Estado de México, Hidalgo, Puebla, Morelos, Tlaxcala, Guerrero, Oaxaca, Veracruz and Chiapas; Guatemala; Honduras; El Salvador; Nicaragua | Mexico-Central America | FP |

| | | | |
|---|---|---|---|
| *P. patula* Schiede ex Schltdl. et Cham. var. *longipedunculata* Lo ock ex Martinez | Mexico: Hidalgo, Veracruz, Oaxaca and Chiapas | Mexico | - |
| *P. patula* Schiede ex Schltdl. et Cham. var. *patula* | Mexico: Tamaulipas, Querétaro, Hidalgo, Estado de México, Ciudad de México, Morelos, Tlaxcala, Puebla, Veracruz, Oaxaca | Mexico | FP |
| *P. pinceana* Gordon | Mexico: Coahuila, Zacatecas, San Luis Potosí, Nuevo León, Querétaro, Hidalgo | Mexico | - |
| **P. praetermissa* Styles et McVaugh | Mexico: Durango, Jalisco, Nayarit, Sinaloa | Mexico | - |
| *P. pringlei* Shaw | Mexico: Michoacán, Guerrero, Estado de México, Morelos, Puebla, Oaxaca | Mexico | FP |
| *P. pseudostrobus* Lindl. var. *apulcensis* (Lindl.) Shaw | Mexico: Chiapas, Guerrero, Hidalgo, Puebla, Tlaxcala, Estado de México, Oaxaca y Veracruz; Guatemala; El Salvador | Mexico-Central America | - |
| *P. pseudostrobus var. coatepecensis* | Mexico: Veracruz, Chiapas, Oaxaca | Mexico | - |
| *P. pseudostrobus* Lindl. var. *pseudostrobus* | Mexico: Chiapas, Guerrero, Hidalgo, Puebla, Tlaxcala, Estado de México, Oaxaca, Veracruz; Guatemala; El Salvador | Mexico-Central America | FP |
| **P. quadrifolia* Parl. ex Sudw | USA: California; Mexico: Baja California | USA-Mexico | - |

| | | | |
|---|---|---|---|
| **P. radiata* D. Don var. *binata* (Engelm.) Lemmon | USA: California; Mexico: Baja California | USA-Mexico | FP |
| **P. remota* (Little) D. K. Bailey et Hawksw | USA: Southwest Texas; Mexico: Northeast and Southeast Chihuahua, Coahuila, and West Nuevo León | USA-Mexico | - |
| *P. rzedowskii* Madrigal et M. Caball. | Mexico: Michoacán, Sierra Madre del Sur | Mexico | - |
| **P. scopulorum* (Engelm.) Lemmon | USA: Montana, North Dakota, South Dakota, Wyoming, Utah, Nevada, Colorado, Nebraska, Oklahoma. New Mexico, Arizona; Mexico: Sonora | USA-Mexico | - |
| **P. strobiformis* Engelm | USA: Arizona, New México, Texas; Mexico: Sonora, Chihuahua, Coahuila, Nuevo León, Durango, Jalisco, Sinaloa, Zacatecas and San Luis Potosí | USA-Mexico | - |
| *P. tecunumanii* Eguiluz et J. P. Perry | Mexico: Oaxaca, Chiapas; Guatemala; Belize; Honduras; El Salvador; and Nicaragua | Mexico-Central America | FP |
| *P. teocote* Schiede ex Schltdl. et Cham | Mexico: Chihuahua, Coahuila, Nuevo León, Tamaulipas, Sinaloa, Durango, Zacatecas, San Luis Potosí, Nayarit, Aguascalientes, Jalisco, Guanajuato, Querétaro, Hidalgo, Michoacán, Estado de México, Ciudad de México, Tlaxcala, Puebla, Veracruz, Guerrero, Oaxaca, Chiapas; Guatemala | Mexico-Central America | FP |
| *P. tropicalis* Morelet | Cuba: Cuba, Mexico | Antilles | FP |
| *P. vallartensis* Pérez de la Rosa & Gernandt | Mexico: Jalisco | Mexico | - |

***Species not entirely distributed within the Neotropical region of Mexico, some with isolated populations, were included due to their ecological importance in the country.**

**Table S2.** Native species of *Pinus* that have been used in forest plantations in Mexico and Central America and introduced in South America.

| Species of *Pinus* in forest plantations in Mexico and Central America | Introduced *Pinus* species in forest plantations in South America |
|---|---|
| *P. ayacahuite* Ehren. | *P. caribaea* |
| *P. caribaea* Morelet | *P. contorta** Douglas ex Loudon |
| *P. cembroides* Zucc. | *P. chiapensis* |
| *P. chiapensis* (Martínez) Adresen | *P. elliottii** Engelm. |
| *P. cooperi* (C.E. Blanco) Farjon. | *P. halepensis*** Mill |
| *P. cubensis* Griseb | *P. lawsonii** Roezl ex Gordon |
| *P. devoniana* Lindl. | *P. leiophylla* |
| *P. douglasiana* Martínez | *P. maximinoi* |
| *P. greggii* Engelm. | *P. oocarpa* |
| *P. hartwegii* Lindl. | *P. pinaster*** Aiton |
| *P. hondurensis* Sénécl. | *P. pinea*** L. |
| *P. jeffreyi* Balf | *P. ponderosa** Lawson & C. Lawson |

| | |
|---|---|
| *P. lawsonii* Roezl. | *P. pringlei* |
| *P. leiophylla* Schl. & Cham. | *P. radiata** D. Don |
| *P. maximartinezii* Rzedowski | *P. taeda** L. |
| *P. maximinoi* H.E. Moore | *P. tecunumani* Eguiluz & J. P. Perry |
| *P. montezumae* Lamb. | |
| *P. occidentalis* Swartz | |
| *P. oocarpa* Schiede | |
| *P. patula* Schl. Et. Cham. | |
| *P. pringei* Shaw. | |
| *P. pseudostrobus* Lindl. | |
| *P. teocote* Schiede ex Schltdl. | |
| *P. tecunumanii* Eguiluz & J.P. Perry | |

*** Pines native to North America **Pine native to Europe**

**Table S3.** Species richness in different taxonomic groups in pine plantations in the neotropical region of South America compared to the natural habitat present.

| Species in Forest Plantation (FP) | Natural Ecosystem (NE) | Taxonomic group | Species total richness (FP) | Species total richness (NE) | Country | Reference |
|---|---|---|---|---|---|---|
| *P. caribaea* | Montane Forest | Plants | 8-20 | 23-37 | Venezuela | [23] |
| *P. contorta* | Pines outside the plantation | ECM Fungi | 4 | 4 | Chile | [72] |
| *P. contorta* | *Nothofagus antarctica* | ECM Fungi | 5 | 8 | Argentina | [36] |
| *P. contorta* | Steppe | Plants | < 20 | 57 | Chile | [29] |
| *P. elliottii* | *Araucaria angustifolia* Forest | Birds | 14 | 17 | Brazil | [33] |
| *P. elliottii* | *Araucaria angustifolia* Forest | Birds | 81 | 93 | Brazil | [73] |
| *P. elliottii* | *Araucaria angustifolia* Forest | Insects | 27 | 22 | Brazil | [35] |
| *P. elliottii* | *Araucaria* sp. Forest | Lepidotera | 33 | 20 | Brazil | [74] |
| *P. elliottii* | *Araucaria angustifolia* Forest | Small mammals | 8 | 10 | Brazil | [75] |
| *P. elliottii* | Tropical Forest | Ants | 60 | 82 | Brazil | [76] |
| *P. elliottii* | Tropical Forest | Macrophyte | 51 | 87 | Brazil | [77] |
| *P. elliottii, P. taeda* | *Araucaria angustifolia* Forest | Anura | 7 | 8 | Brazil | [78] |

| | | | | | | |
|---|---|---|---|---|---|---|
| *P. elliottii, P. taeda* | Grassland | ECM Fungi | 43 | 20 | Argentina | [79] |
| *P. patula* | Paramo | Plants | 14 | 25 | Ecuador | [80] |
| *P. patula* | Paramo | Plants | ≈ 50 | ≈ 75 | Ecuador | [81] |
| *P. patula* | Subparamo | Plants | 76 | 121 | Colombia | [73] |
| *P. patula* | Montane Forests | Plants | 26 | 56 | Colombia | [82] |
| *P. ponderosa* | Xerophytic forest of *Austrocedrus chilensis* | Birds | 8 | 10 | Argentina | [83] |
| *P. ponderosa* | Steppe | Birds | 7 | 10 | Argentina | [83] |
| *P. ponderosa* | *Pinus ponderosa* | ECM fungi | 18 | 157 | Argentina | [9] |
| *P. ponderosa* | SD | ECM fungi | 4 | SD | Argentina | [37] |
| *P. ponderosa* | Xerophytic forest of *Austrocedrus chilensis* | Small mammals | 3 | 7 | Argentina | [84] |
| *P. radiata* | Temperate deciduous forest (*Nothofagus* spp.) | Birds (exotic species) | 1 | 1 | Chile | [30] |
| *P. radiata* | Paramo | ECM fungi | 3 | 0 | Ecuador | [39] |
| *P. radiata* | *Nothofagus glauca* Forest | Insects | 10 | 10 | Chile | [85] |
| *P. radiata* | Temperate deciduous forest (*Nothofagus* spp.) | Insects (exotic species) | 2 | 2 | Chile | [30] |

| | | | | | | |
|---|---|---|---|---|---|---|
| *P. radiata* | Temperate deciduous forest (*Nothofagus* spp.) | Invertebrates | 22 | 27 | Chile | [86] |
| *P. radiata* | Temperate deciduous forest (*Nothofagus* spp.) | Small mammals | 7 | 7 | Chile | [87] |
| *P. radiata* | Temperate deciduous forest (*Nothofagus* spp.) | Small mammals | 7 | 6 | Chile | [35] |
| *P. radiata* | Temperate deciduous forest (*Nothofagus* spp.) | Small mammals | 6 | 9 | Chile | [88] |
| *P. radiata* | Temperate deciduous forest (*Nothofagus* spp.) | Small mammals | 1 | 1 | Chile | [30] |
| *P. radiata* | Temperate deciduous forest (*Nothofagus* spp.) | Plants | 36-48 | 41-57 | Chile | [31] |
| *P. radiata* | Forests of *Nothofagus* Central Zone | Plants | 8 | 31 | Chile | [32] |
| *P. radiata* | Forests of *Nothofagus* Patagonia Zone | Plants | 7 | 16 | Chile | [32] |
| *P. radiata* | Central Zone Scrubland | Plants | 8 | 15 | Chile | [32] |
| *P. radiata* | Central Zone Scrubland | Plants | 7 | 15 | Chile | [32] |
| *P. radiata* | Temperate deciduous forest (*Nothofagus* spp.) | Plants | 146 | 146 | Chile | [89] |
| *P. radiata* | Temperate deciduous forest (*Nothofagus* spp.) | Plants | 53-62 | 73 | Chile | [90] |

| | | | | | | |
|---|---|---|---|---|---|---|
| *P. radiata* | Temperate deciduous forest (*Nothofagus* spp.) | Plants | 15 | 10 | Chile | [30] |
| *P. radiata* | Forests of *Nothofagus obliqua* | Bacteria | 444 | 532 | Chile | [43] |
| *P. radiata* | *Nothofagus glauca, Cryptocaria alba, Laurelia sempervirens, Persea lingue, Nothofagus obliqua* | Bettles | 247 | 251 | Chile | [91] |
| *P. radiata* | Forests of *Nothofagus obliqua* | Fungi | 62 | 74 | Chile | [43] |
| *P. radiata, P. sylvestris* | Forests of *Nothofagus dombeyi* | Birds | 13 | 20 | Chile | [92] |
| *P. radiata, P. sylvestris* | Forests of *Nothofagus dombeyi* | Insects | 34 | 61 | Chile | [92] |
| *P. radiata, P. sylvestris* | Forest of *Nothofagus dombeyi* | Plants | 24 | 42 | Chile | [92] |
| *P. taeda* | Subdeciduous Tropical Forest | Birds | 9 | 18 | Argentina | [93] |
| *P. taeda* | Temperate deciduous forest (*Nothofagus* spp.) | Bettles | 14 | 19 | Argentina | [94] |
| *P. taeda* | Subtropical Forest | ECM Fungi | 7 | 3 | Argentina | [95] |
| *P. taeda* | Subtropical Forestal | Ants | 20 | 36 | Argentina | [96] |
| *P. taeda* | Grassland | Ants | 25 | 22 | Argentina | [96] |
| *P. taeda* | Semi-deciduous Tropical Forest | Mammals | 26 | 30 | Argentina | [93] |

| | | | | | | |
|---|---|---|---|---|---|---|
| *P. taeda, P. elliottii* | ND | ECM Fungi | 15 | ND | Argentina | [38] |
| *Pinus spp.* | ND | ECM Fungi | 33 | ND | Argentina | [39] |
| *Pinus spp.* | Grassland | Plants | 13 | 160 | Brazil | [97] |

**Table S4**. Ectomycorrhizal fungi and bacteria with potential use as inoculants to enhance the physio-morphological development of neotropical pine species of interest for use in forest plantations.

| ECM | Bacteria | Pine host | Inoculum type | Country of origin | Reference |
|---|---|---|---|---|---|
| *Lactarius indigo* | - | *Pinus oocarpa*<br>*Pinus rudis*<br>*Pinus hartwegii*<br>*Pinus pseudostrobus* | Strains obtained from fruiting-bodies | Guatemala | [98] |
| *Hebeloma mesophaeum* | - | *Pinus ayacahuite* | Suspension of spores obtained from dried fruiting-bodies | Mexico | [63] |
| *Laccaria bicolor, Laccaria laccata Hebeloma leucosarx* | | *Pinus greggii* | Suspension of spores obtained from dried fruiting-bodies | Mexico | [69] |
| - | *Enterobacter*<br>*Dyella*<br>*Luteimonas* | *Pinus chiapensis* | Strains isolated from soil | Mexico | [54] |

| | | | | | |
|---|---|---|---|---|---|
| | *Paraburkholderia*<br><br>*Bacillus cepas nativas* | | | | |
| *Astraeus hygrometricus*<br><br>*Pisolithus tinctorius* | - | *Pinus arizonica* | Suspension of spores obtained from fresh fruiting-bodies | Mexico | [99] |
| *Pisolithus tinctorius* *Thelephora terrestris* | - | *Pinus caribaea* var. *hondurensis* | ND | Venezuela | [100] |
| *Laccaria proxima* | *Cohnella* sp commercial strain | *Pinus cembroides* | Suspension of spores obtained from dried fruiting-bodies | Mexico | [55] |
| *Pisolithus tinctorius*<br><br>*Scleroderma texense* | - | *Pinus devoniana*<br><br>*Pinus pseudostrobus* | Powdered inoculum | Mexico | [71] |
| *Astraeus hygrometricus*<br><br>*Boletus barrowsii*<br><br>*Geastrum minum*<br><br>*Russula* cf. | | *Pinus greggii* | Suspension of spores obtained from dried fruiting-bodies | Mexico | [61] |

| | | | | | |
|---|---|---|---|---|---|
| *atroglauca*<br><br>*Suillus caerulescens*<br><br>*Russula xerampelina*<br><br>*Lactarius deliciousus* var. *deterrimus*<br><br>*Russula spp.* | | | | | |
| *Inocybe splendens*<br><br>*Suillus brevipes* | - | *Pinus hartwegii* | Mycelium in liquid BAF medium added to vermiculite and peat | Mexico | [65] |
| *Pisolithus tinctorius* | - | *Pinus pseudostrobus* | Spore suspension + Phosphate fertilization | Mexico | [101] |
| *Laccaria bicolor Hebeloma leucosarx* | - | *Pinus greggii, Pinus montezumae* | Suspension of spores obtained from dried fruiting-bodies | Mexico | [102] |
| *Hebeloma alpinum*<br><br>*Laccaraia trichodermophora*<br><br>*Thelephora* | - | *Pinus pringlei* | Spores from dehydrated caps | México | [45] |

| | | | | | |
|---|---|---|---|---|---|
| *terrestris* | | | | | |
| *Pisolithus microcarpus* | - | *Pinus taeda* | Liquid inoculum Strain CCT4391 | Brasil | [103] |
| - | *Azospirillum brasilense*<br><br>*Bacillus subtilis Pseudomonas fluorescens* | *Pinus taeda* | Liquid inoculum Strain CCT4391 Strain CCTB 03=CNPSo 2719 Strain Ab-V5 and Ab-V6 | Brasil | [60] |
| *Amanita muscaria, Amanita sp., Suillus luteus (Plantation)*<br><br>*Suillus luteus (roots)*<br><br>*Amanita muscaria-Suillus luteus (strains)* | - | *Pinus maximinoi, Pinus patula* | Sporal suspension | Colombia | [67] |
| *Laccaria laccata* | *Cohnella sp.*<br><br>*Azospirillum brasilense* | *Pinus montezumae* | Suspension of spores obtained from dried fruiting-bodies. Bacterial strains in liquid culture medium | Mexico | [13] |
| *Laccaria trichodermophora* | *Pseudomonas fluorescens Pf_ Ag001* | *Pinus montezumae* | Suspension of spores obtained from dried fruiting- | Mexico | [53] |

| | | | | | |
|---|---|---|---|---|---|
| *Laccaria bicolor s.l.*<br><br>*Laccaria vinaceobrunnea*<br><br>*Laccaria laccata* | | | bodies. Commercial bacterial inoculant applied at 0.1 grams per container. | | |
| *Suillus* sp | *Cupriavidus basilensis*<br><br>*Rhodococcus qingshengii*<br><br>*Pseudomonas spp.*<br><br>*Pseudomonas gessardii*<br><br>*Stenotrophomonas rhizophila*<br><br>*Rhodococcus erythropolis*<br><br>*Cohnella sp.*<br><br>*Obtained from the rhizosphere of Pinus hartwegii, Abies and. Cupressus, P.* | *Pinus pseudostrobus* | Mycelium strain CPEc1 Bacterial suspension | Mexico | [52] |

| | | | | | |
|---|---|---|---|---|---|
| | *montezumae* | | | | |
| *Rhizopogon luteolus*<br><br>*Suillus*<br><br>*granulatus* | - | *Pinus radiata* | Strains obtained from a mature plantation of *P. radiata* | Chile | [21] |
| *Boletus edulis*<br><br>*Boletus pinicola* | - | *Pinus radiata* | Sporal suspension from lyophilized fruiting-bodies. | Chile | [104] |
| *Russula luteolus*<br><br>*Suillus luteus* | | *Pinus radiata* | Suspension of spores obtained from dried fruiting-bodies. | Chile | [105] |
| *Amanita muscaria*<br><br>*Suillus luteus*<br>*Amanita sp.* | - | *Pinus patula*<br><br>*Pinus oocarpa*<br><br>*Pinus tecunumanii* | Sporal suspension from fresh fruiting-bodies of a *P. patula* plantation | Colombia | [106] |
| *Suillus luteus*<br><br>*Rhizopogon roseolus, Hebeloma mesophaeum*<br><br>Forestal soil | - | *Pinus ponderosa* | Sporal suspension from fresh fruiting-bodies of a *P. ponderosa* plantation | Argentina | [62] |

| | | | | | |
|---|---|---|---|---|---|
| *Suillus luteus*<br><br>*Hebeloma mesophaeum*<br><br>*Rhizopogon roseolus*<br>*Tricholoma muricatum* | - | *Pinus ponderosa* | Sporal suspension from dried fruiting-bodies of adjacent plantations | Argentina | [68] |
| *Hebeloma alpinum*<br><br>*Hebeloma leucosarx*<br><br>*Hebeloma mesophaeum* | - | *Pinus patula*<br><br>*Pinus pseudostrobus* | Sporal suspension from dried fruiting-bodies | Mexico | [107] |
| *Amanita muscaria*<br>*Suillus luteus* | | *Pinus patula* | Sporal suspension | Colombia | [67] |
| *Amanita stranella*<br><br>*Suillus decipiens* | | *Pinus pseudostrobus* var. *coatepecensis* | Sporal suspension | Mexico | [66] |

**Table S5**. Inoculants based on mycorrhizae and bacteria suggested and used in forest plant production that are marketed in Mexico

| Product | Origin | Composition | Cost | Yield |
|---|---|---|---|---|
| Ectospor | Mexico | *Pisolithus tinctorius, Rhizopogon amylopogon, R. bilosuli, R. fulvigleba, R. luteolus, Lacaria bicolor, L. laccata, Scleroderma citrini* 11,200,000 spores/g<br><br>*Trichoderma harzianum, T. reesei* 1,500,000 CFU/g (1.5x$10^6$)<br><br>*Azospirillum brasiliense, Azotobacter chroococcum, Bacillus megaterium, Pseudomonas fluorescens* 1,000,000 CFU/g (1x$10^6$) | $420.77 USD/kg | 1kg/200,000 seedlings |
| MycoRacine | Mexico | *Pisolithus tinctorius* 525 x $10^5$ spores/gr | $19.95 USD/kg | 1kg/3000-9000 seedlings |
| PHC® Mycor Tree® Ecto-Inyectable® | Mexico | *Pisolithus tinctorius* 7.8 millon spores/g, *Scleroderma citrinum* 780,000 spores/g, rhizobacteria, and yucca plant extracts. | $61 USD/227 g | 227 grams in 20 liters of water to inoculate 15,000 seedlings or 60,000 seedlings per kilogram of dry product. |
| PHC® Ecto-Rhyza® | Mexico | *Pisolithus tinctorius* (Pt) 250 million UFC/g and antagonistic fungi *Trichoderma harzianum* Strain T-22 (KRL-AG2) 5×$10^4$ CFU/g | $230.78 USD/kg | 1 Kg/100,000 seedlings |
| PHC® MycorTree® Endo y Ecto® Inyectable | Mexico | *Pisolithus tinctorius* 890 million spores per bag.<br><br>*Glomus clarum, Glomus intraradices, Glomus etunicatum, Entrophospora columbiana* 80,000 spores per packet of product.<br><br>12 billion rhizobacteria, humic acids, formononetin, and | $55.13 USD/ Bag containing 2 packets of 113 grams each | One packet in 100 liters of water and apply one liter per established tree or 250 milliliters per seedling in the nursery. |

| | | | | |
|---|---|---|---|---|
| | | nutrients. | | |
| Micorrizas Grow Depot | México | *G. intraradices, G. mosseae, G. brasilianum, G. clarum, G. deserticola, G. etunicatum, Gigaspora margarita.*<br><br>*Azosporillum brasilense, Azotobacter chroococcum, Bacillus megaterium* and *Pseudomonas fluorescens.* | $10.89 USD/kg | 50 gr/ L of water<br><br>1 L = 25 seedlings |
| EctoPlant | España | *Rhizopogon* spp, *Pisolithus tinctorius, Scleroderma verrucosum, Suillus* spp | $45.94 USD/500 tablets | 1 tablet per plant |
| Burize ST® Buckman Laboratories, S.A de C.V. | México | *Glomus intraradices* | ND | 2.4 grams of BuRize® per liter. 20 milliliters of the solution per plant |
| MycoGrow® Micronized Endo/Ecto Seed Mix | USA | *Rhizopogon fulvigleba, R. villosullus, R. luteolus, R. amylopogon* (2,750 propagules)<br><br>*Pisolithus tinctorius* (220,509 propagules/g)<br><br>*Scleroderma* strain, *S. citrinum* (5,500 propagules/doses) | $7.62 USD/28 g | 113 a 300 g each 0.7 $m^3$ of soil. |
| Bio Bravo® | Mexico | *Azospirillum brasilense*, *Glomus* spp, *Trichoderma* spp.<br><br>$2x10^6$ CFU/doses, 18000 spores, $1x10^7$ spores/doses | $42.08 USD/500 ml | 500 milliliters per square meter of substrate (in seedbed). |
| Ecto-Myc® | Mexico | *Amanita rubescens, Amanita sp., Lactarius indigo, Ramaria* sp., *Boletus* sp. (10x $10^6$ spores/ml). | ND | 1.5 liters per 1,000 liters of water. Substrate application: In germination trays of 77 cavities of 170 ml of base mix. Irrigation |

| | | | | |
|---|---|---|---|---|
| | | | | application: Apply 10 ml of broth per plant or 1,000 liters of broth for 100,000 plants. |
| Astuto | Mexico | *Bacillus thuringiensis* var. *kurstaki* strain ABTS-351<br><br>32,000 CFU/mg | $48.02 USD/kg | 0.6 kg/1.5 Ha |

The costs presented are approximate and may vary depending on market conditions.
